# Functional Analysis of the Ser149/Thr149 Variants of Human Aspartylglucosaminidase and Optimization of the Coding Sequence for Protein Production

**DOI:** 10.3390/ijms18040706

**Published:** 2017-03-26

**Authors:** Antje Banning, Jan F. König, Steven J. Gray, Ritva Tikkanen

**Affiliations:** 1 Institute of Biochemistry, Medical Faculty, University of Giessen, Friedrichstrasse 24, 35392 Giessen, Germany; Antje.Banning@biochemie.med.uni-giessen.de (A.B.); jan.friedrich.koenig@googlemail.com (J.F.K.); 2 Gene Therapy Center and Department of Ophthalmology, University of North Carolina, Chapel Hill, NC 27302, USA; Steven_Gray@med.unc.edu

**Keywords:** aspartylglucosaminidase, lysosomal storage disorder, aspartylglucosaminuria, lysosomes, single nucleotide polymorphism, gene therapy

## Abstract

Aspartylglucosaminidase (AGA) is a lysosomal hydrolase that participates in the breakdown of glycoproteins. Defects in the *AGA* gene result in a lysosomal storage disorder, aspartylglucosaminuria (AGU), that manifests mainly as progressive mental retardation. A number of AGU missense mutations have been identified that result in reduced AGA activity. Human variants that contain either Ser or Thr in position 149 have been described, but it is unknown if this affects AGA processing or activity. Here, we have directly compared the Ser149/Thr149 variants of AGA and show that they do not differ in terms of relative specific activity or processing. Therefore, Thr149 AGA, which is the rare variant, can be considered as a neutral or benign variant. Furthermore, we have here produced codon-optimized versions of these two variants and show that they are expressed at significantly higher levels than AGA with the natural codon-usage. Since optimal AGA expression is of vital importance for both gene therapy and enzyme replacement, our data suggest that use of codon-optimized AGA may be beneficial for these therapy options.

## 1. Introduction

**Figure 1:**
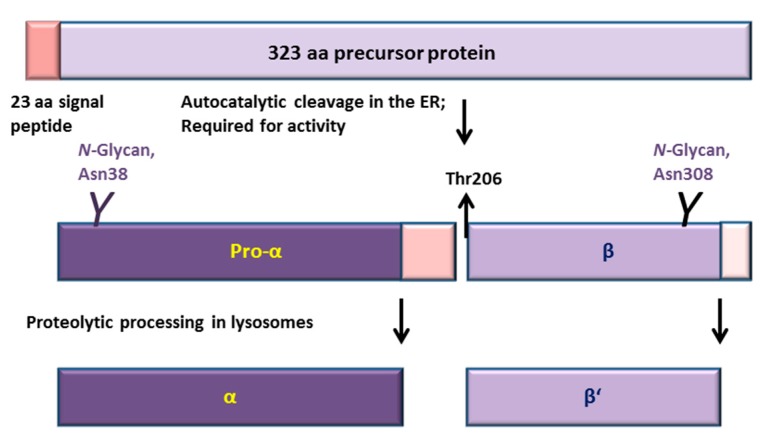
Processing and activation of aspartylglucosaminidase (AGA). AGA is synthesized in the ER as a 346 amino acid (aa) polypeptide from which 23 residues of the signal peptide are removed. AGA contains two *N*-glycosylation sites at Asn38 and Asn308 [5]. Very soon after synthesis in the ER, two AGA precursors homodimerize, inducing an autocatalytic cleavage of both precursors N-terminally to Thr206 into 27 kDa pro-α and 17 kDa β subunits. After transport to lysosomes, the pro-α is C-terminally cleaved into 24 kDa mature α subunit, whereas processing of the β subunit gives rise to the 14 kDa β’ subunit. Neither of these lysosomal processing steps displays an effect on the enzyme activity.

Aspartylglucosaminidase (AGA, *N*4-(β-*N*-Acetylglucosaminyl)-l-Asparaginase, EC 3.5.1.26) is a lysosomal hydrolase that participates in one of the final steps during the degradation of *N*-glycosylated proteins. AGA cleaves the bond between *N*-acetylglucosamine and asparagine after the polypeptide backbone has been degraded [1]. AGA is synthesized as a single-chain precursor molecule that soon after synthesis in the endoplasmic reticulum (ER) homodimerizes and becomes processed into two pro-α and β subunits (Figure 1) [2]. This autocatalytic cleavage takes place between the residues Asp205 and Thr206 and is a prerequisite for the activity of the enzyme [3,4]. After transport into lysosomes, both subunits are C-terminally trimmed. The final active form of AGA is a heterotetramer of two α and two β or β’ subunits (α_2_β_2_) [5]. AGA belongs to the group of so-called N-terminal nucleophile (NTN) hydrolases, as the free α-amino group of Thr206 is involved in the catalysis as the base, whereas the OH group of Thr206 functions as a nucleophile during the catalysis [3,6]. The members of the NTN hydrolase family, which in addition to AGA also include, e.g., the proteasome β subunit and penicillin acylase, show very little similarity at the amino acid sequence level, but they exhibit a highly similar folded structure [6].

Mutations in the *AGA* gene result in aspartylglucosaminuria (AGU, OMIM 208400), a lysosomal storage disorder that is characterized by progressive loss of intellectual capabilities and some skeletal abnormalities [7,8,9]. AGU patients are born seemingly normal, but the progressive course of the disease manifests in, e.g., developmental delay, loss of speech and coarse facial features early in childhood [10]. In adulthood, most AGU patients are severely retarded and require special care. AGU is a rare disease with an unknown prevalence in most populations, but it is enriched in the Finnish population [7,11]. Due to a founder effect, a specific gene defect designated as AGU_Fin-major_ is found in homozygous form in most Finnish AGU patients, although the parents do not show any consanguinity [12,13]. The AGU_Fin-major_ mutation, which is a combination of two missense mutations, results in an exchange of Cys163 to Ser, abolishing a disulfide bond and destabilizing the AGA structure [12,14]. This pathogenic substitution is always combined with a functionally neutral Arg161Gln substitution [12,14]. The second most common allele in Finland is a 2 bp deletion called AGU_Fin-minor_ (NM_000027.3; c.199_200del - p.Glu67fc*3) [15]. Outside Finland, most patients have their individual mutations, either in homozygous form, when originating from consanguineous marriages, or as compound heterozygous mutations [16,17,18].

AGU mutations result in reduced AGA activity in patient cells. However, depending on the mutation type and its consequences on the AGA protein expression, the degree of residual enzyme activity may vary considerably [19]. Severe consequences on AGA expression are observed in the case of deletions, insertions and splicing mutations, which basically abolish AGA protein expression. Very low AGA activities are also detected in the case of nonsense mutations, whereas missense mutations show considerable heterogeneity in terms of residual activity [19]. Our recent findings have shown that in the case of some point mutations, including AGU_Fin-major_ and Thr122Lys, the amino acid changes have moderate consequences on the AGA enzyme structure, and these mutant forms can be converted into an active form by means of pharmacological chaperones that stabilize the enzyme structure [20].

A large majority of the AGU mutations, including AGU_Fin-major_, reside outside the active site of the enzyme. The Thr122Lys substitution in turn resides in a loop structure close to the interface of the two halves of the tetrameric AGA, and is also predicted to cause a local folding defect without any severe effect on AGA expression levels [20]. The only AGU mutation that hits the active site of AGA is Ser72Pro substitution that causes aberrant processing of the AGA precursor [21]. However, Ser72 does not directly participate in the catalysis, but is hydrogen-bonded to the catalytic Thr206 [3,6]. Interestingly, patients exhibiting this mutation show considerable residual enzyme activity and appear to exhibit a milder disease phenotype. These findings show that it is important to understand the consequences of amino acid substitutions on AGA structure in order to make predictions on AGA activity.

**Figure 2:**
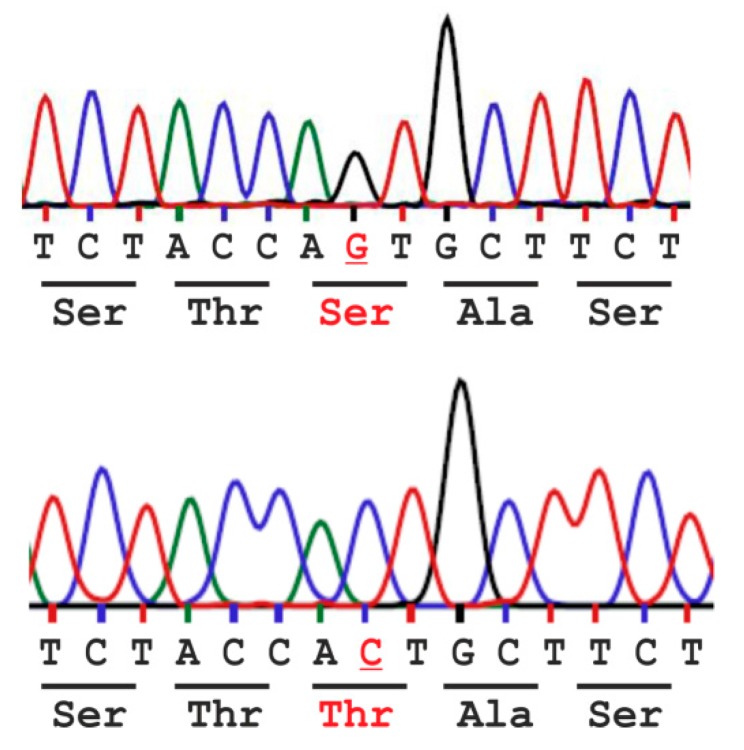
Single nucleotide polymorphism rs2228119 (NM_000027.3:c.446C>G - p.(Thr149Ser) results in amino acid variation Ser vs. Thr at position 149 (base and amino acid variation in red) of the human AGA enzyme.

The human *AGA* genomic sequence contains a missense single nucleotide polymorphism (SNP) rs2228119 (NM_000027.3:c.446C>G - p.Thr149Ser) at amino acid position 149, encoding either for ACT/Thr or AGT/Ser (Figure 2). It is known from various genetic databases that these variants exist, but so far, it has not been characterized if these variants exhibit any differences in terms of processing or activity of AGA. Although the Thr149 variant is clearly the rarer one (see Section 2.1), the official *AGA* reference sequence NM_000027.3 contains the Thr149 variant. Since most scientists starting to work on *AGA* or AGU intuitively would use the reference sequence, it is important to characterize the possible differences in activity and processing between these two variants. Furthermore, since both gene therapy approaches and enzyme replacement therapy (ERT) for AGU are currently under development, it will be of great importance to compare these natural variants in terms of their activity. In addition to these analyses, we have here tested the expression of the codon-optimized Ser/Thr149 variants of human AGA. We here show that Ser149 and Thr149 are highly similar in terms of AGA processing and relative specific activities. However, the Thr149 variant shows a mildly higher expression level in overexpression systems. The codon-optimized variants of AGA show significantly higher protein and activity levels upon overexpression in human cells than the natural human variants. Therefore, our data suggest that codon optimization of AGA may be useful for both gene therapy and ERT.

## 2. Results

### 2.1. Analysis of the Population Frequency of a Natural AGA Variant NM_000027.3:c.446C>G - p.Thr149Ser

Variant frequency and distribution for NM_000027.3:c.446C>G - p.Thr149Ser were deduced from the “1000 genomes project” [22,23] which provides sequence data from 2504 individuals originating from five continental regions and 26 different populations. Worldwide, the allele frequency for Ser149 was 92%, and 86.3% of all tested subjects were homozygous for the Ser149 variant, while 11.5% were heterozygous and only 2.2% were homozygous for Thr149. However, looking at the global distribution, most Thr149 alleles are found in African populations (allele frequency: Ser 71%, Thr 29%), with most of the Thr149 positive probands being heterozygous for Ser149 and Thr149. In most European, South and East Asian populations, the Ser149 variant accounts for 100% of the alleles. The only exception within Europe is the Iberian population in Spain where 0.9% of the sequenced genomes contained the Thr variant in a heterozygous manner. Remarkably, in the Finnish population, which has the world’s highest AGU prevalence, Ser149 was found to be the sole genotype, which was also confirmed by the sequencing data of the Exome Aggregation Consortium that contain the genomes of 3307 Finnish individuals (See http://exac.broadinstitute.org/variant/4-178359960-G-C) [24]. Table 1 summarizes the distribution of allele frequencies among various populations.

### 2.2. Consequences of the Ser/Thr149 Variants on the Activity and Processing of the AGA Enzyme

**Figure 3:**
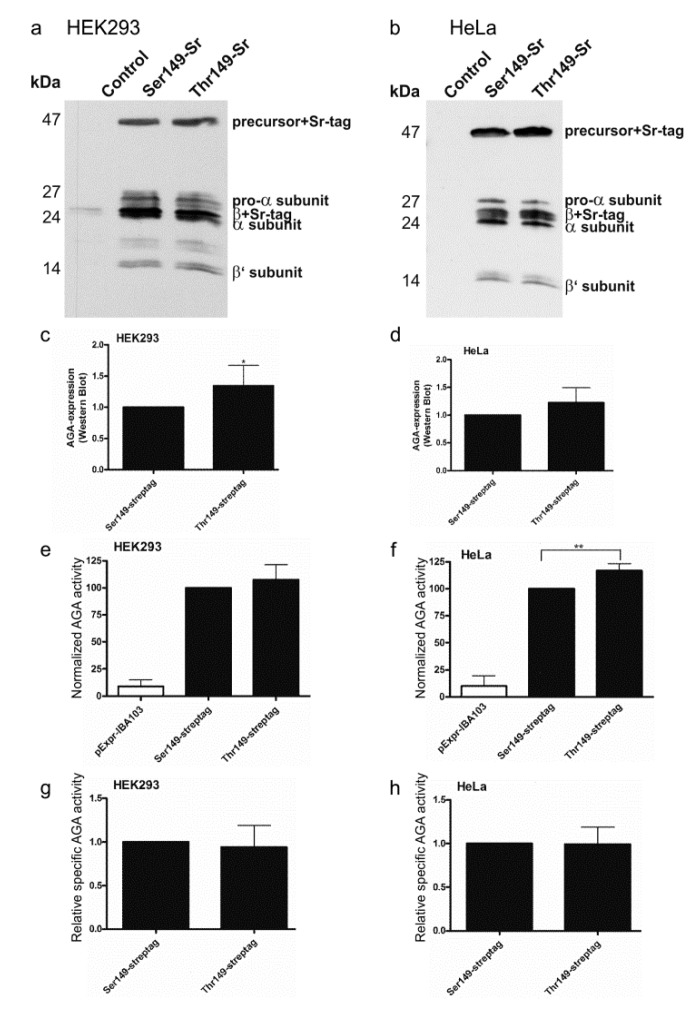
Processing and activity of Strep-tagged AGA variants. SNP149-AGA variants with C-terminal Twin-strep-tag were transiently expressed in (**a**) HEK293T or (**b**) HeLa cells. Empty pExpr-IBA103 served as control. Western Blot with anti-AGA antibody shows correct processing of all constructs; (**c**,**d**) Western Blot signals were quantified and normalized to renilla luciferase activity to correct for transfection efficiency; (**e**,**f**) AGA activity was measured in the same cell lysates as used for Western Blot and normalized to renilla luciferase activity; (**g**,**h**) AGA activity was normalized to AGA protein amount. *n* ≥ 4, shown as mean ± SD. Statistical analysis by unpaired *t*-test (**c**,**d**,**g**,**h**) and One-Way ANOVA (**e**,**f**). Values of *p* < 0.05 were considered significant (*) and values of *p* < 0.01 very significant (**).

To check if the Ser/Thr variants exhibit differences in the activity or processing, the Thr substitution was introduced into the Ser149 construct in pEXPR-IBA103 [20]. The resulting AGA variants are thus expressed as Twin-Strep-tagged fusion proteins. We have shown that the tagged AGA becomes processed into subunits and exhibits normal enzyme activity [20]. The AGA variants were expressed in HEK93T and HeLa cells and analyzed by Western Blot using anti-AGA antibodies (Figure 3a–d). Both Ser149 and Thr149 AGA variants became processed into subunits and exhibited virtually identical patterns of bands in HEK (Figure 3a) and HeLa cells (Figure 3b). The Western Blot signals were quantified and normalized for the transfection efficiency using cotransfected renilla luciferase activity as a measure. In both HEK and HeLa cells, the Thr149 variant exhibited a slightly higher expression level than the Ser149 AGA. Consistent with a higher expression level, the Thr149 also showed a higher level of activity (again normalized for transfection efficiency as above) especially in HeLa cells (Figure 3e,f). However, the relative specific activities (activity correlated with protein level) did not significantly differ from each other (Figure 3g,h).

### 2.3. Expression of Codon-Optimized Variants of Ser149 and Thr149 AGA

**Figure 4:**
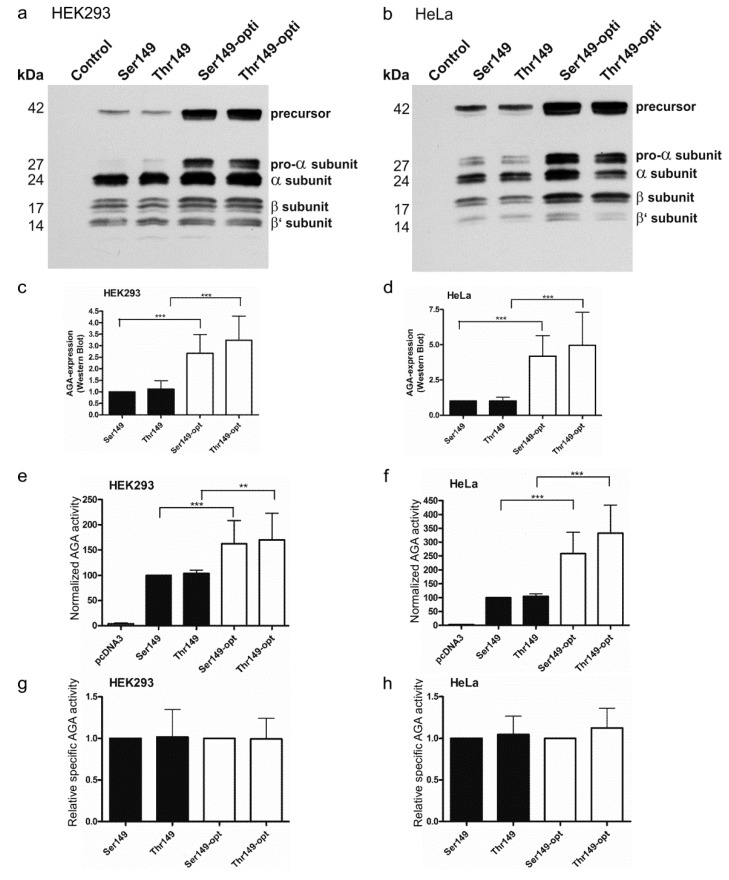
Comparison of processing and activity of the optimized and natural AGA variants. SNP149-AGA variants were transiently expressed in (**a**) HEK293T or (**b**) HeLa cells. Empty pcDNA3 plasmid served as a control. Western Blot with anti-AGA antibody shows correct processing of all constructs, with higher expression level of codon-optimized constructs; (**c**,**d**) Western Blot signals were quantified and normalized to renilla luciferase activity to correct for transfection efficiency; (**e**,**f**) AGA activity was measured in the same cell lysates as used for Western Blot. AGA activity was normalized to renilla luciferase activity; (**g**,**h**) AGA activity was normalized to AGA protein amount. *n* = 5, shown as mean ± SD. Statistical analysis by One-Way ANOVA. Values of *p* < 0.01 were considered very significant (**) and *p* < 0.001 extremely significant (***).

AGU is a lysosomal storage disorder for which gene or enzyme replacement therapies (ERT) are potential treatment options. In both cases, it is important to obtain a high level of enzyme expression and activity in the target tissues. Increased expression of the AGA enzyme might be obtained by codon optimization of the constructs used for gene therapy or production of the recombinant enzyme for ERT. We thus optimized the codon usage of the human *AGA* gene and cloned the optimized Ser149 and Thr149 variants in pcDNA3 expression vector. In these optimized versions, 217 of the 346 codons in AGA were exchanged for the optimal ones for human expression. The expression and activity of the optimized variants were compared with constructs exhibiting the normal human cDNA sequence. Renilla luciferase activity was again used to correct for the differences in the transfection efficiencies. A significantly higher protein expression was obtained in HEK and HeLa cells for the codon optimized variants, as compared to the respective non-optimized ones (Figure 4a–d). Consistently, the normalized enzyme activities were significantly higher with the optimized AGA variants (Figure 4e,f). The optimized Thr149 AGA again showed a tendency to higher expression and activity than the Ser149 counterpart, but this difference was not significant. As with the non-tagged AGA variants, the relative specific activities of Ser149 and Thr149 AGA were not significantly different from each other (Figure 4g,h).

### 2.4. Expression of AGA Variants in Patient Fibroblasts

**Figure 5:**
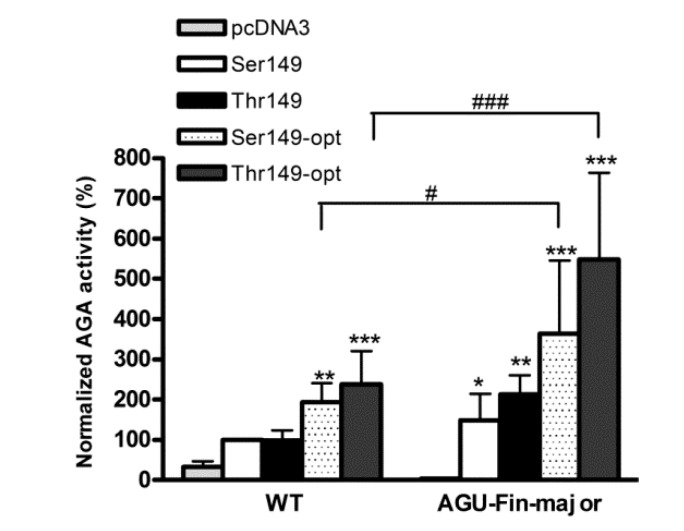
Activity of the optimized and natural AGA variants in control and patient fibroblasts. The Thr/Ser149 AGA variants were transiently expressed in wildtype (WT) and AGU_Fin-major_ (AGU-Fin) fibroblasts. The pcDNA3 plasmid without insert served as a control. AGA activity was measured and normalized to total protein amount of the lysates. Normalized AGA activity in wildtype fibroblasts transfected with the natural AGA variant Ser149 was set as 100%. *n* = 4, shown as mean ± SD. Statistical analysis by Two-Way ANOVA. * vs. pcDNA3, # vs. WT. Values of *p* < 0.05 were considered significant (* or **#**) while values of *p* < 0.01 were considered very significant (**) and *p* < 0.001 extremely significant (*** or **###**).

Codon-optimized protein variants may provide a higher level of protein expression and thus activity, which would be beneficial for gene therapy approach. However, it has been shown by numerous studies that the use of synonymous codons may alter mRNA structure or even folding and posttranslational modifications of some proteins (reviewed in [25]). As this may severely affect the outcome of therapies based on codon-optimized proteins, we tested if the optimized AGA variants would result in higher enzyme activities when transfected in patient fibroblasts. The resulting AGA activities were measured and normalized to total protein amount in the lysates. Due to the low transfections rates, normalization with renilla luciferase (see above) was not feasible. Our data show that transfection of both wildtype and AGU_Fin-major_ fibroblasts with the optimized AGA variants resulted in significantly higher enzyme activities as compared to the natural variants (Figure 5). Interestingly, we consistently observed significantly higher AGA activities in the transfected AGU_Fin-major_ fibroblasts than in the respective wildtype ones. This is most likely due to the stabilizing effect of the cotransfected wildtype AGA (natural or optimized) on the endogenous, mutated AGU_Fin-major_ polypeptide, as we have shown previously [20].

**Figure 6:**
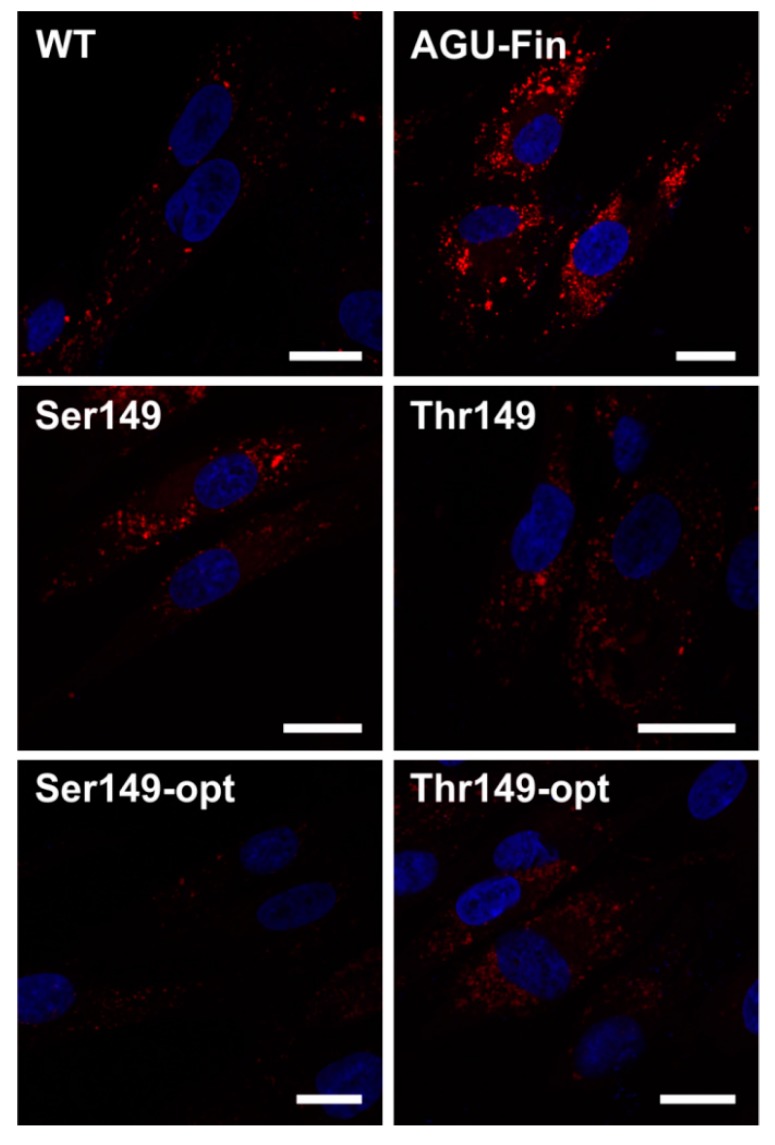
Transfection with the optimized and natural AGA variants in aspartylglucosaminuria (AGU) patient fibroblasts improves lysosomal morphology. The Thr/Ser149 AGA variants were transiently expressed in AGU_Fin-major_ (AGU-Fin) fibroblasts. **Uppermost** row: WT and AGU-Fin fibroblasts transfected with pCDNA3; **Middle** and **lowermost** row: AGU-Fin fibroblasts transfected with the indicated constructs. The cells were stained with Lysotracker-Red and the nuclei were visualized with 4′,6-diamidin-2-phenylindol (DAPI). Scale bar 20 µm.

To show that the transfection with the *AGA* variants also improves the lysosomal morphology in AGU fibroblasts, we stained control-transfected wildtype (WT) and AGU_Fin-major_ fibroblasts transfected with the *AGA* variants with Lysotracker to visualize lysosomes (Figure 6). Control-transfected AGU_Fin-major_ cells showed highly enlarged lysosomes. However, transfection with the *AGA* variant constructs clearly improved lysosomal morphology in these cells. Unfortunately, due to the low transfection efficiency, the data could not be quantified.

## 3. Discussion

We have here characterized the consequences of the genomic variant NM_000027.3:c.446C>G - p.(Thr149Ser) in the human *AGA* gene that results in variants of AGA enzyme carrying Ser vs. Thr in position 149. So far, it has been unclear if the Thr variant represents a benign or pathogenic substitution that may affect the processing or the activity of the enzyme. In most populations, the Ser variant is the predominant or even the only one, but especially in some African populations, the Thr variant is relatively common.

We were able to show that these two variants do not differ from each other in terms of processing or activity. The Thr149 AGA showed a slightly higher expression level especially in HeLa cells. The reasons for the higher expression level of Thr149 AGA upon overexpression are not clear, but this variant might, e.g., exhibit a slightly longer half-life than the Ser149 AGA. However, the relative specific activities (enzyme activity correlated to AGA protein amount) of Ser149 and Thr149 AGA were highly similar. This suggests that this variant does not directly affect the enzyme activity, which is consistent with the fact that the residue 149 does not participate in enzyme catalysis, nor does it reside within the active site of AGA. Furthermore, the autocatalytic activation by processing into the subunits appears to take place equally well for both variants, suggesting that the folding is not directly affected, in accordance with the similar character and size of Thr and Ser residues.

The observed higher expression level of the Thr149 AGA at the first glimpse suggests that this variant might be the better option when considering gene therapy or enzyme production for ERT. However, some caution needs to be called for, since the Thr149 AGA is only observed in specific populations, such as some African and South-American ones. Therefore, it is possible that immunological responses against the Thr149 AGA might be observed in patients that naturally carry the Ser149 variant. In this study, we also generated codon-optimized versions of Ser149 and Thr149 AGA. The optimized AGA enzymes showed the same tendency for a higher expression level of the Thr149 variant, but no difference in the relative specific activity, as observed for the enzymes synthesized upon standard codon usage. Thus, we conclude that the Thr149 variant does not differ from the Ser149 AGA in terms of specific activity, and should thus be considered as a benign variant that does not exhibit any functional impairment.

The codon-optimized AGA enzyme variants exhibited 2.5- to 5-fold higher expression in HEK and HeLa cells, as compared to the AGA enzymes with standard codons. These data show that optimization of the codon usage can indeed result in significantly higher expression of AGA in heterologous expression systems. Optimization of protein expression is an important aspect when considering the production of AGA enzyme for ERT. In addition, it is also important that high expression levels of AGA are obtained by gene therapy approaches to facilitate the spread of the active enzyme from the cells carrying the gene therapy construct to the neighboring cells and even to cells farther away in the tissues. However, the potential adverse effects that may result from the altered codon usage should be considered [25,26]. Numerous studies have addressed the effect of synonymous or optimized codons on protein expression (excellently reviewed in [25]). Although such codon substitutions may result in faster protein translation and thus higher expression levels, it has been shown that they may also adversely affect, e.g., the secondary structure and stability of the mRNA and thus result in unexpectedly low protein yields. Even more interestingly, “rare” codons that are translated more slowly appear to be located in areas of the protein that may require more time for proper folding. Thus, replacing these codons with “fast ones” may impair protein folding during synthesis and result in a non-functional protein. Thus, it is important to test the effect of codon optimization on the protein activity before using this strategy for, e.g., gene therapy.

Especially in the case of proteins like AGA that undergo numerous processing steps, care should also be taken not to overload the expression system. Our data show that upon high degree of AGA expression, especially with the optimized variants, the AGA precursor form starts to accumulate. Therefore, it is important to ensure that on the one hand, maximum production of the active AGA is obtained, but the expression system is not choked by expression levels that are too high, which would result in production of the inactive AGA precursor. For enzyme production for ERT, expression systems primed for very high level production should thus be used to obtain a maximum amount of active AGA enzyme.

We have here tested both the natural and the codon-optimized *AGA* gene constructs in AGU patient fibroblasts that are homozygous for AGU_Fin-major_. We showed that expression of both the natural and the optimized versions of AGA resulted in higher enzyme activities, with the optimized variants producing the highest activity. Intriguingly, transfection of AGU_Fin-major_ fibroblasts resulted in an AGA activity that was consistently even higher than that obtained with the transfected WT fibroblasts. Although we cannot completely exclude an effect of different transfection efficiencies between WT and AGU_Fin-major_ cells, we postulate that this positive effect may also be due to the ectopic WT AGA precursor acting as a “folding aid” for the endogenous AGU polypeptides. The processing of AGA precursors into the active subunit form requires that two precursor molecules dimerize. We have earlier shown that cotransfection of WT and AGU_Fin_ constructs results in processing and activation of the mutant AGA precursor that remains as a precursor in the absence of any WT protein [20]. This is most likely due to the fact that in the cotransfected (or here: WT *AGA* transfected) cells, the WT precursors associate with the AGU_Fin_ ones and are able to support their folding and processing, thus resulting in a high degree of improvement of AGA activity in the patient cells. Unfortunately, the low sensitivity of the available AGA antibodies did not allow us to test this hypothesis by Western Blot. However, such a WT-aided processing of the endogenous, mutant AGA precursor would greatly facilitate gene therapy in AGU patients, as even a lower degree of expression of the WT protein would result in a relatively high correction of enzyme activity, and most likely also of the lysosomal pathology.

## 4. Materials and Methods

### 4.1. Analysis of the Genomic Variant NM_000027.3:c.446C>G - p.Thr149Ser

Worldwide distribution and frequency of the genomic variant NM_000027.3:c.446C>G - p.Thr149Ser (SNP rs2228119) was analyzed with the “1000 Genomes” browser (http://www.1000genomes.org/ensembl-browser), which uses genotyping data of 2504 genomes from 26 populations [23].

### 4.2. Cell Culture

HeLa (human cervix carcinoma cells, (Cat. CCL-2, ATCC, Wesel, Germany), and HEK293T (human embryonic kidney cells, Cat. CRL-3216, ATCC,) cells were cultured in Dulbecco’s modified Eagle’s medium (DMEM, Gibco, Thermo Fisher Scientific, Karlsruhe, Germany) with high glucose, supplemented with 10% fetal calf serum (FCS, Gibco), 100 U/mL Penicillin and 100 µg/mL Streptomycin (Sigma-Aldrich, Taufkirchen, Germany) at 8% CO_2_ and 37 °C. Primary skin fibroblasts with AGU-Fin mutation were obtained from Coriell Institute of Medical Research (Catalog ID: GM00568; Camden, NJ, USA). Immortalized normal human skin fibroblasts were as described [27]. Fibroblasts were cultured as described [20].

### 4.3. Plasmids

AGA Ser149 variant constructs in pcDNA3 (Invitrogen, Thermo Fisher Scientific) and pExpr-IBA103 (IBA, Göttingen, Germany) vectors were as described [20]. These constructs served as templates for mutagenesis of Ser149 into Thr149. Codon-optimization and synthesis of the human *AGA* sequence carrying the Thr149 was carried out by DNA2.0 (Menlo Park, CA, USA), cloned into pJ600 (DNA2.0), and subcloned into pcDNA3. The full sequence for the codon-optimized *AGA* is available from the authors upon request. The resulting construct served as template for mutagenesis of Thr149 into Ser149. Correctness of all constructs was verified by sequencing. The cloning primer sequences were:

AGA-pcDNA3-fwd: 5′-CTATAGGATCCATGGCGCGGAAGTCGAACTTG-3′;
AGA-pcDNA3-rev: 5′-CTATACTCGAGTTAGATGCAGTCCACTTTTTCC-3′;
AGA-IBA-fwd: 5′-CTATATCTAGAATGGCGCGGAAGTCGAACTTG-3′;
AGA-IBA-rev: 5′-CTATAGGATCCGATGCAGTCCACTTTTTCCTC-3′;
optiAGA-pcDNA3-fwd: 5′-CTATAGGATCCATGGCACGCAAGTCAAACCTC-3′;
optiAGA-pcDNA3-rev: 5′-CTATACTCGAGTTAGATGCAATCGACCTTCTCC-3′.

### 4.4. Transfection

Twenty-four hours prior to transfection, the cells were seeded onto 12-well plates. For transfections, 450 ng of the respective *AGA* expression plasmid and 50 ng of pRL-TK encoding renilla luciferase for normalization (Promega, Mannheim, Germany) were transfected using MACSfectin™ (Miltenyi Biotec, Bergisch Gladbach, Germany) according to the manufacturer’s protocol. After 24 h, the cells were transferred into 6-well plates and harvested 48 h post-transfection using 250 µL passive lysis buffer (Promega). Fibroblasts were electroporated using the Neon™ Transfection System (Invitrogen) according to the manufacturer’s instruction. 1 × 10^6^ cells in a total volume of 100 µL were transfected with 1 µg plasmid (1200 V, 40 ms, 1 pulse). Cells that were used for lysotracker stainings were cotransfected with 250 ng pEGFP-N1 plasmid (Clontech, Takara, Saint-Germain-en-Laye, France) for the detection of transfected cells. Transfected cells were either seeded into 6 well plates and used for enzyme activity measurements or onto coverslips and used for staining with Lysotracker as described [20].

### 4.5. Enzyme Activity Measurements

AGA activity was measured fluorimetrically as described in [20]. For the normalization of the transfection efficiencies in Hela and HEK cells, renilla luciferase activity was measured with a Tecan infinite M200 plate reader using 20 µL of 20-fold diluted lysate and 85 µL of renilla juice (PJK, Kleinblittersdorf, Germany). AGA activity was divided by renilla activity to compensate for the variations in transfection efficiencies. For the transfected fibroblasts, activities were correlated with the protein amount.

### 4.6. Western Blotting Analysis

Equal volumes of non-diluted lysates were analyzed by 15% SDS-polyacrylamide gel electrophoresis and Western Blot. For antibody staining, the membranes were blocked with 5% non-fat dry milk in TBST (10 mM Tris, 150 mM NaCl, 0.05% Tween 20) and incubated overnight at 4 °C with a rabbit antiserum against the α and β subunits of AGA [5] in TBST. This was followed by incubation with HRP-conjugated secondary antibodies in TBST for 1 h at room temperature before development with Western Blotting Detection Reagents.

### 4.7. Statistical Analysis

All experiments were performed at least four times. Data are expressed as mean ± SD. Statistical comparisons between groups were made using Student’s *t*-tests, One-Way or Two-Way ANOVA, as appropriate (GraphPad Prism 5, GrapdPad Software Inc., La jolla, CA, USA). Western Blot bands were quantified by scanning densitometry using Quantity One Software (version 4.6, Bio-Rad, Munich, Germany) and normalized against the Renilla luciferase activity measured from the same lysates (see above). Values of *p* < 0.05 were considered significant (*) while values of *p* < 0.01 were considered very significant (**) and *p* < 0.001 extremely significant (***).

## Figures and Tables

**Table 1:** Genotype frequency of single nucleotide polymorphism (SNP) rs2228119 in various populations.

Genotype	Ser/Ser (%)	(*n*)	Ser/Thr (%)	(*n*)	Thr/Thr (%)	(*n*)
All Populations Studied	86.3	(2162)	11.5	(288)	2.2	(54)
**Europe**	**99.8**	**(502)**	**0.2**	**(1)**	-	-
Finnish in Finland	100	(99)	-	-	-	-
British in England and Scotland	100	(91)	-	-	-	-
Iberian populations in Spain	99.1	(106)	0.9	(1)	-	-
Toscani in Italy	100	(107)	-	-	-	-
Utah residents with Northern and Western European ancestry	100	(99)	-	-	-	-
**North and South America**	**94.8**	**(329)**	**5.2**	**(18)**	-	-
Colombian in Medellin	94.7	(89)	5.3	(5)	-	-
Mexican Ancestry in Los Angeles	100	(64)	-	-	-	-
Peruvian in Lima, Peru	98.8	(84)	1.2	(1)	-	-
Puerto Rican in Puerto Rico	88.5	(92)	11.5	(12)	-	-
**Africa**	**51.1**	**(338)**	**40.7**	**(269)**	**8.2**	**(54)**
African Caribean in Barbados	62.5	(60)	32.3	(31)	5.2	(5)
African Ancestry in Southwest US	57.4	(35)	39.3	(24)	3.3	(2)
Esan in Nigeria	46.5	(46)	44.4	(44)	9.1	(9)
Luhya in Webuye, Kenya	46.5	(46)	44.4	(44)	9.1	(9)
Mandinka in Gambia	46.9	(53)	39.8	(45)	13.3	(15)
Mende in Sierra Leone	48.2	(41)	41.2	(35)	10.6	(9)
Yoruba in Ibadan, Nigeria	52.8	(57)	42.6	(46)	4.6	(5)
**East Asia**	**100**	**(504)**	**-**	**-**	**-**	**-**
Chinese Dai in Xishuangbanna	100	(93)	-	-	-	-
Han Chinese in Bejing	100	(103)	-	-	-	-
Southern Han Chinese	100	(105)	-	-	-	-
Japanese in Tokyo, Japan	100	(104)	-	-	-	-
Kinh in Ho Chi Minh City, Vietnam	100	(99)	-	-	-	-
**South Asia**	**100**	**(489)**	**-**	**-**	**-**	**-**
Bengali in Bangladesh	100	(86)	-	-	-	-
Gujarati Indian in Houston, Tx	100	(103)	-	-	-	-
Indian Telegu in the UK	100	(102)	-	-	-	-
Punjabi in Lahore, Pakistan	100	(96)	-	-	-	-
Sri Lankan Tamil in the UK	100	(102)	-	-	-	-

## Abbreviations

aa	Amino acid
AGA	Aspartylglucosaminidase
AGU	Aspartylglucosaminuria
DAPI	4′,6-Diamidin-2-phenylindol
DMEM	Dulbecco’s modified Eagle’s medium
ER	Endoplasmic reticulum
ERT	Enzyme replacement therapy
FCS:	Fetal calf serum
HEK293T	Human embryonic kidney cells
HeLa	Human cervix carcinoma cells
NTN	N-terminal nucleophile
SNP	Single nucleotide polymorphism

## References

[1] Makino M., Kojima T., Yamashina I. (1966). Enzymatic cleavage of glycopeptides. Biochem. Biophys. Res. Commun..

[2] Ikonen E., Julkunen I., Tollersrud O.K., Kalkkinen N., Peltonen L. (1993). Lysosomal aspartylglucosaminidase is processed to the active subunit complex in the endoplasmic reticulum. EMBO J..

[3] Oinonen C., Tikkanen R., Rouvinen J., Peltonen L. (1995). Three-dimensional structure of human lysosomal aspartylglucosaminidase. Nat. Struct. Biol..

[4] Wang Y., Guo H.C. (2003). Two-step dimerization for autoproteolysis to activate glycosylasparaginase. J. Biol. Chem..

[5] Tikkanen R., Enomaa N., Riikonen A., Ikonen E., Peltonen L. (1995). Intracellular sorting of aspartylglucosaminidase: The role of N-linked oligosaccharides and evidence of Man-6-*P*-independent lysosomal targeting. DNA Cell Biol..

[6] Tikkanen R., Riikonen A., Oinonen C., Rouvinen R., Peltonen L. (1996). Functional analyses of active site residues of human lysosomal aspartylglucosaminidase: Implications for catalytic mechanism and autocatalytic activation. EMBO J..

[7] Palotie L., Ikonen E., Syvanen A.C., Halila R., Enomaa N., Heiskanen T., Gron K., Aula P. (1991). Molecular genetics of aspartylglucosaminuria. Duodecim.

[8] Pollitt R.J., Jenner F.A., Merskey H. (1968). Aspartylglycosaminuria. An inborn error of metabolism associated with mental defect. Lancet.

[9] Autio S. (1972). Aspartylglycosaminuria. Analysis of thirty-four patients. J. Ment. Defic. Res..

[10] Autio S., Visakorpi J.K., Jarvinen H. (1973). Aspartylglycosaminuria (AGU). Further aspects on its clinical picture, mode of inheritance and epidemiology based on a series of 57 patients. Ann. Clin. Res..

[11] Mononen T., Mononen I., Matilainen R., Airaksinen E. (1991). High prevalence of aspartylglycosaminuria among school-age children in eastern Finland. Hum. Genet..

[12] Ikonen E., Baumann M., Gron K., Syvanen A.C., Enomaa N., Halila R., Aula P., Peltonen L. (1991). Aspartylglucosaminuria: cDNA encoding human aspartylglucosaminidase and the missense mutation causing the disease. EMBO J..

[13] Mononen I., Heisterkamp N., Kaartinen V., Williams J.C., Yates J.R., Griffin P.R., Hood L.E., Groffen J. (1991). Aspartylglycosaminuria in the Finnish population: Identification of two point mutations in the heavy chain of glycoasparaginase. Proc. Natl. Acad. Sci. USA.

[14] Ikonen E., Enomaa N., Ulmanen I., Peltonen L. (1991). In vitro mutagenesis helps to unravel the biological consequences of aspartylglucosaminuria mutation. Genomics.

[15] Isoniemi A., Hietala M., Aula P., Jalanko A., Peltonen L. (1995). Identification of a novel mutation causing aspartylglucosaminuria reveals a mutation hotspot region in the aspartylglucosaminidase gene. Hum. Mutat..

[16] Ikonen E., Peltonen L. (1992). Mutations causing aspartylglucosaminuria (AGU): A lysosomal accumulation disease. Hum. Mutat..

[17] Opladen T., Ebinger F., Zschocke J., Sengupta D., Ben-Omran T., Shahbeck N., Moog U., Fischer C., Burger F., Haas D. (2014). Aspartylglucosaminuria: Unusual neonatal presentation in Qatari twins with a novel aspartylglucosaminidase gene mutation and 3 new cases in a Turkish family. J. Child. Neurol..

[18] Saarela J., Laine M., Oinonen C., von Schantz C., Jalanko A., Rouvinen J., Peltonen L. (2001). Molecular pathogenesis of a disease: Structural consequences of aspartylglucosaminuria mutations. Hum. Mol. Genet..

[19] Saarela J., Laine M., Tikkanen R., Oinonen C., Jalanko A., Rouvinen J., Peltonen L. (1998). Activation and oligomerization of aspartylglucosaminidase. J. Biol. Chem..

[20] Banning A., Gulec C., Rouvinen J., Gray S.J., Tikkanen R. (2016). Identification of small molecule compounds for pharmacological chaperone therapy of aspartylglucosaminuria. Sci. Rep..

[21] Peltola M., Tikkanen R., Peltonen L., Jalanko A. (1996). Ser72Pro active-site disease mutation in human lysosomal aspartylglucosaminidase: Abnormal intracellular processing and evidence for extracellular activation. Hum. Mol. Genet..

[22] Genomes Project C., Auton A., Brooks L.D., Durbin R.M., Garrison E.P., Kang H.M., Korbel J.O., Marchini J.L., McCarthy S., McVean G.A. (2015). A global reference for human genetic variation. Nature.

[23] Sudmant P.H., Rausch T., Gardner E.J., Handsaker R.E., Abyzov A., Huddleston J., Zhang Y., Ye K., Jun G., Hsi-Yang Fritz M. (2015). An integrated map of structural variation in 2504 human genomes. Nature.

[24] Lek M., Karczewski K.J., Minikel E.V., Samocha K.E., Banks E., Fennell T., O’Donnell-Luria A.H., Ware J.S., Hill A.J., Cummings B.B. (2016). Analysis of protein-coding genetic variation in 60,706 humans. Nature.

[25] Shabalina S.A., Spiridonov N.A., Kashina A. (2013). Sounds of silence: Synonymous nucleotides as a key to biological regulation and complexity. Nucleic Acids Res..

[26] Mauro V.P., Chappell S.A. (2014). A critical analysis of codon optimization in human therapeutics. Trends Mol. Med..

[27] Mussche S., Devreese B., Nagabhushan Kalburgi S., Bachaboina L., Fox J.C., Shih H.J., van Coster R., Samulski R.J., Gray S.J. (2013). Restoration of cytoskeleton homeostasis after gigaxonin gene transfer for giant axonal neuropathy. Hum. Gene Ther..

